# Traditional uses of wild and tended plants in maintaining ecosystem services in agricultural landscapes of the Eastern Cape Province in South Africa

**DOI:** 10.1186/s13002-022-00512-0

**Published:** 2022-03-15

**Authors:** Alfred Maroyi

**Affiliations:** Department of Botany, University of Fort Hare, Private Bag X1314, Alice, 5700 South Africa

**Keywords:** Eastern Cape Province, Ecosystem services, Plant biodiversity, Traditional ecological knowledge, Useful plants

## Abstract

**Background:**

Many communities in developing countries rely on ecosystem services (ESs) associated with wild and cultivated plant species. Plant resources provide numerous ESs and goods that support human well-being and survival. The aim of this study was to identify and characterize wild and tended plant species, and also investigate how local communities in the Eastern Cape Province in South Africa perceive ESs associated with plant resources.

**Methods:**

The study was conducted in six local municipalities in the Eastern Cape Province, between March 2016 and September 2021. Data on socio-economic characteristics of the participants, useful plants harvested from the wild and managed in home gardens were documented by means of questionnaires, observation and guided field walks with 196 participants. The ESs were identified using a free listing technique.

**Results:**

A total of 163 plant species were recorded which provided 26 cultural, regulating and provisioning ESs. Provisioning ESs were the most cited with at least 25 plant species contributing towards generation of cash income, food, traditional and ethnoveterinary medicines. Important species recorded in this study with relative frequency of citation (RFC) values > 0.3 included *Alepidea amatymbica*, *Allium cepa*, *Aloe ferox*, *Artemisia afra*, *Brassica oleracea*, *Capsicum annuum*, *Cucurbita moschata*, *Hypoxis hemerocallidea*, *Opuntia ficus-indica*, *Spinacia oleracea*, *Vachellia karroo* and *Zea mays*.

**Conclusion:**

Results of this study highlight the importance of plant resources to the well-being of local communities in the Eastern Cape within the context of provision of essential direct and indirect ESs such as food, medicinal products, construction materials, fodder, regulating, supporting and cultural services. The ESs are the basis for subsistence livelihoods in rural areas, particularly in developing countries such as South Africa. Therefore, such body of knowledge can be used as baseline data for provision of local support for natural resource management initiatives in the province and other areas of the country.

## Background

A growing body of literature suggests that local communities in developing countries still continue to rely heavily on provisioning ecosystem services (ESs) derived from plants around their homesteads for their basic necessities [1–6]. Research by Bidak et al. [2] showed that plant resources provide direct ESs such as serving as sources of food, medicines, energy and shelter. There are also indirect social and economic ESs derived from plant resources. Ecosystem services are classified into four categories: provisioning ESs such non-timber forest products, fibre, firewood, water, fish and natural medicines; regulating ESs such as pollination, natural hazards, water, air quality, runoff, disease and climate regulation; cultural services that provide recreational, aesthetic and spiritual benefits; and supporting ESs such as habitat for species, soil formation, photosynthesis and nutrient cycling [7]. Plant resources supply all three major categories of ESs, that is, provisioning, regulating and cultural services, including the supporting services that enable agricultural landscapes to be productive [8, 9]. Plant resources provide ESs that extend beyond the provision of food, medicines, energy, shelter and some of these ESs are indirect, unmanaged, underappreciated and undervalued. For example, research by Ladino et al. [10] showed that the plant group Bromeliads provide direct benefits to the human society, and they also form microecosystems in which accumulated water and nutrients support the communities of aquatic and terrestrial species. Previous research by Calvet-Mir et al. [8] and Camps-Calvet et al. [9] showed that plants grown and managed in home gardens are important for the provision of cultural ESs. The authors argued that plant resources managed in home gardens offer nature-based solutions to environmental problems, protection and restoration of agricultural landscapes, promotion of healthy lifestyles, social integration and environmental justice. Similarly, Barrios et al. [11] argued that tree-mediated ESs are recognized as key features of more sustainable agroecosystems but the strategic management of tree attributes for ES provision is poorly understood. For example, Barrios et al. [11] opined that managing trees for ESs requires understanding tree identities, their characteristics, uses and how to manage these trees in the provision of all four ESs categories in different socio-ecological contexts. Despite their importance and everyday use, comprehensive knowledge of the ecology and socio-economic value of ESs derived from plant resources is largely lacking, hindering the ability to monitor, regulate and manage them. Therefore, a clear understanding about the condition of provisioning, regulating, cultural and supporting services provided by plant resources is necessary and such information is derived from both the resource use patterns of the people who are most reliant on these services, as well as the utility of the plant resources exploited by local communities.

Mensah et al. [3] argued that ESs underpin human livelihoods around the world as the use of ESs is important for decision-making processes that target the social expectations of local communities. However, a detailed understanding of the complex variation in the use and relative importance of ESs at the household level is required to fully understand how ESs affect livelihoods across different landscapes [12–15]. Other researchers are of the view that determination and identification of ESs is a prerequisite in order to estimate the relative importance of ESs in a community and ensuring their conservation and sustainable management [16–18]. Therefore, understanding the linkages between ESs and benefits for people is critical for safeguarding natural resources and particularly those important for groups that are most vulnerable to global change [19, 20]. However, the ability of ESs to inform decision-making has been limited by knowledge gaps about the links between ES supply and the delivery and distribution of benefits [20–25]. Despite growing interest in ESs provided by plant biodiversity in different landscapes [26–30], few studies have attempted to systematically describe and evaluate the ESs derived from wild and tended plant species. Current statistics show that most ESs research has focused on higher income countries [31] while growing body of literature shows that there is direct dependence on ESs in low-income countries [32, 33]. Therefore, the aim of this study was to assess ESs supplied by wild and tended plant species in the Eastern Cape Province in South Africa. The specific goals of this paper are: i. to identify and characterize the diversity of wild and tended plant species, and ii. to investigate how local communities in the Eastern Cape Province perceive ESs associated with plant resources.

## Materials and methods

### Study area

**Fig. 1:**
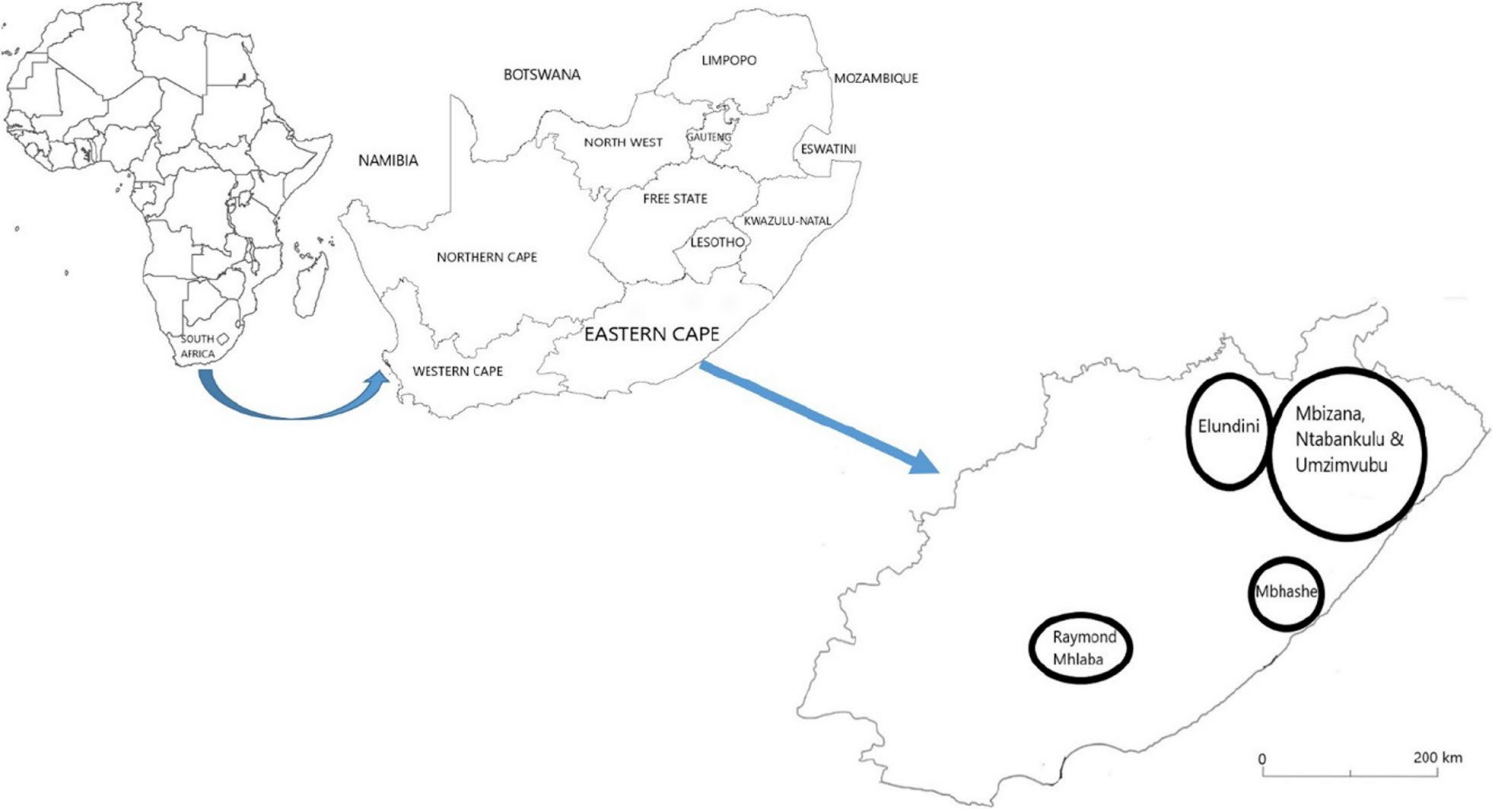
Map of South Africa illustrating the geographical position of the study areas

This study was conducted in six local municipalities in the Eastern Cape Province in South Africa, namely Elundini, Mbhashe, Mbizana, Ntabankulu and Raymond Mhlaba and Umzimvubu (Fig. 1). The Eastern Cape Province is the second largest province in the country covering 168 966 km^2^ of land area [34]. It is regarded as a rural province and is predominantly inhabited by isiXhosa speaking people of Cape Nguni descent. The Eastern Cape Province includes two of the former homeland areas, namely Ciskei and Transkei out of the thirteen former racially-defined homelands or Bantustan areas of South Africa [35]. One of the Apartheid government’s acts of segregation was the Bantu Authorities Act of 1951, which legalized the deportation of Black people into designated homelands. Black people were forcibly removed from urban areas and white farms to those areas demarcated as homelands. The Ciskei and Transkei are today characterized by landlessness, pervasive chronic poverty, low levels of education, economic activity, vulnerability, lack of basic services, a dearth of employment opportunities and high levels of dependency on welfare [36, 37]. An estimated 72% of the population in the Eastern Cape Province live below the poverty line, which is more than the national average of 60% and this is attributed to the legacies of Apartheid, where the Eastern Cape provincial administration inherited the largely impoverished and corrupt former Ciskei and Transkei homelands [38]. Research by Westaway [37] revealed that the majority of households in the Eastern Cape Province spend most of their income on food and there is clear evidence of growing food insecurity as measured by the number of meals consumed and the quantity and variety of foods eaten. Most people in the province live in rural areas, the contribution of agriculture to local livelihoods is low in the entire province and has been in decline for several decades [39]. Research by Shackleton et al. [40] revealed that local people’s livelihoods in the province are centred on grasslands and forests for fodder, wild foods, firewood, medicinal plants and fibre species for weaving.

The six local municipalities are in the Savanna Biome [41] dominated by grassland, succulent thicket and *Acacia* thornveld, and species such as *Aloe aborescens* Mill., *Aloe ferox* Mill., *Diospyros dichrophylla* (Grand.) De Winter, *Eragrostis curvula* (Schrad.) Nees, *Euphorbia* spp., *Melinis nerviglumis* (Franch.) Zizka, *Olea europaea* L. ssp. *africana* (Mill.) P. S. Green and *Vachellia karroo* (Hayne) Banfi & Glasso. The altitude of the study area is between 0 and 1860 m above sea level, characterized by summer rainfall and dry frosty winters with approximately 500–1069 mm per year [42, 43]. Mean maximum and minimum monthly temperatures are 38 °C in summer and 4 °C in winter, respectively [42–44]. The Eastern Cape Province is characterized by a variety of land uses ranging from commercially oriented rangeland stock farming to small-scale vegetable and crop production [42]. Other economic activities in the province include tourism, forestry and wool production [43]. Major crops cultivated in the study area include beetroot (*Beta vulgaris* L.), cabbage (*Brassica oleracea* L.), carrots (*Daucas carota* L.), maize (*Zea mays* L.), potatoes *(Solanum tuberosum* L.) and spinach (*Spinacia oleracea* L.). The majority of the inhabitants (at least 87%) in the study sites are traditional isiXhosa speaking people who are highly dependent on natural resources for their livelihoods [45].

### Data collection

This study is part of a wider research on utilization of plant resources and other non-timber forest products in the Eastern Cape Province [46–52]. Therefore, sampling occurred during one week field excursions conducted between March 2016 and September 2021. Sampling was carried out in six Local Municipalities (Fig. 1). Standardized plant sampling procedures were used to collect specimens [53, 54] involving transect walks in home gardens, farms and the surrounding landscapes. Interview discussions were conducted in the local language, isiXhosa and were translated into English with the help of an interpreter. During the interviews, we documented information on names of plant species, uses, plant parts used and preparation of useful plants. The ESs were identified using a free listing technique [10, 55], whereby interviewees were asked to list benefits and contributions of plant species to human well-being. The stated benefits were matched with the four major categories in the established ES classifications which included provisioning ESs (timber and firewood, edible plants, edible fruits and medicinal plants), regulating ESs (benefits from natural and ecological processes, e.g. pest control, pollination), supporting ES (healthy soil) and cultural ESs (tourism and recreation) [7, 56]. These categories were expanded or modified based on information obtained during fieldwork, focus group discussions and field observations. Plant species were identified in the field and the taxon names conform to those of Germishuizen et al. [57] and the Plants of the World Online [58]. Unknown plant species were collected, pressed, oven-dried and identified by taxonomists at the Giffen Herbarium (UFH) at the University of Fort Hare and Schonland Herbarium (GRA) at Rhodes University, Grahamstown, South Africa.

Ethnobotanical data were gathered from 196 purposively sampled participants using snowball sampling technique [59–61]. The participants were requested to sign University of Fort Hare (MAR011) informed consent form and researchers also adhered to the ethical guidelines of the International Society of Ethnobiology (www.ethnobiology.net). The majority of these participants (55.6%) were males and their age ranged from 18 to 84 years. More than half of the participants (57.7%) were above 50 years, while 34.2% were below 40 years of age. About half of the sample (46.4%) were single, 23.5% were married, 16.8% and 13.3% were widowed and divorced, respectively. Close to half of the sample (44.4%) were educated up to primary level, while 21.9% of the sample were educated up to secondary level, 17.9% and 15.8% of the sample had attained tertiary education or no formal education, respectively. About half of the participants (48.5%) were unemployed, surviving on government social grants and remittances.

### Data analysis

The data collected were entered in Microsoft Excel 2016 file and this data were used to determine frequencies and other descriptive statistical patterns. Interview responses from participants were coded and sorted into themes, paying particular attention to inconsistencies and unique statements. The relative frequency of citation (RFC) of reported plant species was determined using the following equation:

$${\text{RFC}} = {\text{FC/}}N\left( {0 < {\text{RFC}} < 1} \right)$$

This index shows the local importance of each species and is given by the frequency of citation (FC) that is the number of informants mentioning the use of species divided by the total number of informants participating in the study [62, 63]

## Results and discussion

### Floristic composition

A total of 163 plant species were recorded in the Eastern Cape Province (Table 1), with herbs, shrubs and trees having the most species. Pteridophytes and gymnosperms were represented by a single species each, that is, *Cheilanthes hirta* Sw. (family Sinopteridaceae) and *Podocarpus latifolius* (Thunb.) R.Br. ex Mirb. (family Podocarpaceae), respectively. A large number of the plant species (67.5%, *n* = 110) recorded in this study are from 16 families (Table 2). The other 29 families had less representation, between one and two species each. Plant families with the highest number of species were: Asteraceae (16 species), Poaceae (14), Fabaceae (10 species), Solanaceae (nine species), Amaryllidaceae (eight species), Asphodelaceae (seven species), Apiaceae (six species), Amaranthaceae, Asparagaceae, Cactaceae, Myrtaceae and Rosaceae (five species each), Apocynaceae, Lamiaceae and Myrtaceae (four species each) and Salicaceae (three species) (Table 2). Bennett [64] categorized seven of these plant families, that is, Apiaceae, Fabaceae, Lamiaceae, Malvaceae, Myrtaceae, Poaceae and Solanaceae as vital for human existence. Bennett [64] also argued that diets of most cultures around the world rely substantially on species of Fabaceae and Poaceae families, and the world’s three most important cultivated plants, that is, *Oryza sativa* L. (rice), *Triticum aestivum* L. (wheat) and *Zea mays* L. (maize) are grasses and belong to the Poaceae family. Similarly, Hammer and Khoshbakht [65] argued that Asteraceae, Fabaceae and Poaceae are among the most important families in the world as these families have high numbers of domesticated and semi-domesticated species. Among the recorded genera, those species belonging to *Acacia* Martius, *Aloe* L., *Asparagus* Tourn. ex L., *Bulbine* Wolf, *Helichrysum* Mill., *Opuntia* Mill., *Prunus* L., *Solanum* L. and *Tulbaghia* L. with three species each were the dominant taxa. From the 163 plant species recorded, 96 (58.9%) were indigenous to South Africa while 67 species (41.1%) were exotic (Table 1). Similar results were obtained by Akinnifesi et al. [66] who recorded 40.0% exotic plant species in home gardens of São Luis in Brazil arguing that such exotic species ensures biodiversity and genetic diversity. Other researchers argue that exotic species introduced purposefully offer economic and intrinsic benefits when used as food plants [67–69], fuel [67, 70–72], herbal medicines [73–75], ornamental [76–78] and shelter [67, 79, 80]. Similarly, Kahane et al. [81] and Caballero-Serrano et al. [82] argued that indigenous and traditional plant species are usually less attractive to farmers and some commercial exotic species are preferred as these species are easier to grow and more marketable than indigenous and traditional food plants. Evaluation of plant diversity in home gardens of Kerala in India revealed that exotic plants constituted 51.0% of the recorded species [83].

**Table 1:** Diversity and use categories of plant species in the Eastern Cape Province

Species name and voucher number	Family	Vernacular name	Growth form	Parts used	Uses	RFC
*Acacia baileyana F. Muell.; AM1421	Fabaceae	Iwatlisi	Tree	Stems	Construction timber	0.03
*Acacia dealbata Link.; AM1422	Fabaceae	Idywabasi	Tree	Stems	Construction timber	0.04
*Acacia mearnsii De Wild.; AM1424	Fabaceae	Idywabasi	Tree	Bark, leaves, stems and twigs	Construction timber, firewood, fodder, windbreak and sold as herbal medicine (diarrhoea)	0.07
*Acokanthera oblongifolia* (Hochst.) Codd; AM1451	Apocynaceae	Intlungunye and inxinebe	Tree	Leaves	Sold as herbal medicine (ethnoveterinary medicine (EVM), headache and snakebite)	0.09
*Agapanthus africanus* Hoffmanns; AM1432	Amaryllidaceae	Isicakathi	Herb	Leaves and whole plant	Ornamental and herbal medicine (antiseptic, rash and stomach problems)	0.05
*Agave americana L.; AM1452	Asparagaceae	Ikhamanga	Shrub	Leaf sap and whole plant	Fibre, live fence, ornamental and herbal medicine (high blood pressure)	0.06
*Alepidea amatymbica* Eckl. & Zeyh.; AM1494	Apiaceae	Iqwili	Herb	Roots	Sold as herbal medicine (abdominal pains, fever, pimples and wounds)	0.33
*Alepidea serrata* Eckl. & Zeyh*.*; AM1505	Apiaceae	Ubulawa	Herb	Roots	Herbal medicine (cough)	0.01
*Allium cepa L.; AM1507	Amaryllidaceae	Itswele	Herb	Bulb	Edible tubers and bartered with neighbours or sold in local markets	0.41
*Allium sativum L.; AM1508	Amaryllidaceae	Ivimbampunzi	Herb	Bulb	Edible tubers and herbal medicine (cough)	0.12
*Aloe arborescens* Mill.; AM1493	Asphodelaceae	Ikhala and ingcelwane	Shrub	Leaf gel and leaves	Herbal medicine (EVM, constipation, dry skin, inflammation, stomach problems and wounds)	0.12
*Aloe ferox* Mill.; AM1409	Asphodelaceae	Ikhala and umhlaba	Shrub	Leaf gel, leaves, stems and whole plant	Firewood, live fence, animal enclosure and sold as herbal medicine (EVM, boils, dry skin, immune booster, stomachache, tuberculosis(TB) and wounds); leaf gel harvested and marketed	0.38
*Aloe greatheadii* Schönland var. *davyana*. (Schönland) Glen & D.S. Hardy; AM1559	Asphodelaceae	Inkala	Herb	Leaf gel and leaves	Herbal medicine (burns, sores and wounds)	0.01
*Aloiampelos ciliaris* (Haw.) Klopper & Gideon F.Sm. var. *ciliaris*; AM1506	Asphodelaceae	Intelezi	Shrub	Leaf gel and leaves	Herbal medicine (wounds)	0.01
*Amaranthus hybridus L.; AM1515	Amaranthaceae	Nomdlomboyi, umfino, umtyutyu and unomdlomboyi	Herb	Leaves	Leafy vegetable	0.04
*Amaranthus spinosus* L.; AM1560	Amaranthaceae	Utyuthu	Herb	Leaves	Leafy vegetable	0.06
*Artemisia afra* Jacq*.* ex Willd.; AM1516	Asteraceae	Umhlonyane	Shrub	Leaves and roots	Herbal medicine (cough, diabetes, loss of appetite and TB)	0.32
*Arundo donax L.; AM1561	Poaceae	Intsasela	Grass	Whole plant	Thatching grass and erosion control	0.01
*Asparagus africanus* L.; AM1495	Asparagaceae	Ubulawu, ubumhlope, umathunga and umthunzi	Climber	Leaves and roots	Sold as herbal medicine (sexually transmitted Infections (STIs), to speed up labour and wounds)	0.14
*Asparagus asparagoides* (L.) Druce; AM1433	Asparagaceae	Imvane and isicakathi	Climber	Roots	Herbal medicine (STIs)	0.01
*Asparagus laricinus* Burch.; AM1562	Asparagaceae	Inqatha	Shrub	Roots	Herbal medicine (EVM, inflammation, TB and wounds)	0.01
*Avena fatua L.; AM1563	Poaceae	Ihabile	Grass	Whole plant	Fodder	0.01
*Bambusa balcooa Roxb. ex Roxb.; AM1564	Poaceae	Ingcongolo	Grass	Whole plant	Thatching grass	0.01
*Beta vulgaris L.; AM1489	Amaranthaceae	Bhetruthi	Herb	Leaves and tuber	Edible tubers and leaves, and bartered with neighbours or sold in local markets	0.11
*Bidens pilosa L.; AM1536	Asteraceae	Umhlabangubo, umhlabangulo, ucadolo and uqadolo	Herb	Leaves	Leafy vegetable and herbal medicine (TB)	0.04
*Boophone disticha* (L. f.) Herb.; AM1496	Amaryllidaceae	Incwadi and ishwadi	Herb	Bulb	Sold as herbal medicine (EVM, boils and circumcision wounds)	0.13
*Bowiea volubilis* Harv. ex Hook. f. ssp. *volubilis;* AM1490	Asparagaceae	Umagaqana, umagaquana and umgaqana	Herb	Bulb	Sold as herbal medicine (headache, inflammations and impotence)	0.13
*Brassica oleracea L.; AM1535	Brassicaceae	Ikhaphetshu	Herb	Leaves	Leafy vegetable and bartered with neighbours or sold in local markets	0.64
*Brassica spp.; AM1534	Brassicaceae	–	Herb	Leaves	Leafy vegetable and bartered with neighbours or sold in local markets	0.03
*Bromus catharticus Vahl; AM1565	Poaceae	Irhasi	Grass	Whole plant	Fodder	0.01
*Bruguiera gymnorrhiza* (L.) Lam.; AM1533	Rhizophoraceae	Isiqungati	Tree	Stems	Construction timber	0.04
*Bulbine abyssinica* A. Rich.; AM1492	Asphodelaceae	Intelezi and uyakayakana	Herb	Leaves and roots	Sold as herbal medicine (EVM, diarrhoea and menstrual problems)	0.05
*Bulbine frutescens* (L.) Willd.; AM1532	Asphodelaceae	Ibhucu	Herb	Leaf sap	Herbal medicine (EVM, diabetes, ringworm and wounds)	0.09
*Bulbine latifolia* (L. f.) Roem. & Schult.; AM1531	Asphodelaceae	Ibhucu and incelwane	Herb	Roots	Sold as herbal medicine (EVM, diarrhoea and speed up labour)	0.15
*Caesalpinia decapetala (Roth) Alson; AM1566	Fabaceae	Bobo	Shrub	Fruits, stems and whole plant	Edible fruits, firewood, animal enclosure and live fence	0.02
*Cannabis sativa L.; AM1567	Cannabaceae	Intsanga, somntsangu and umya	Shrub	Leaves	Smoked for pleasure and sold as herbal medicine (EVM, diabetes, epilepsy, headache, labour pains and respiratory infections)	0.02
*Capparis tomentosa* Lam.; AM1410	Capparaceae	Imfishlo, intshihlo, intsihlo and umpasimani	Tree	Roots	Herbal medicine (pneumonia, snakebite and sore throat)	0.06
*Capsicum annuum L.; AM1447	Solanaceae	Itshilisi	Herb	Fruits	Edible fruits, herbal medicine (fever) and bartered with neighbours or sold in local markets	0.35
*Carissa bispinosa* (L.) Desf. ex Brenan; AM 448	Apocynaceae	Beta-umtumzi	Shrub	Fruits	Edible fruits	0.04
*Carpobrotus edulis* (L.) L. Bolus; AM1449	Aizoaceae	Igcukuma	Shrub	Leaves	Herbal medicine (ringworm, sore throat, TB and wounds)	0.08
*Catha edulis* (Vahl) Endl.; AM1568	Celastraceae	Igqwaka, iqwaka and umhlwazi	Tree	Leaves	Leafy vegetable	0.01
*Catharanthus roseus (L.) G. Don; AM1450	Apocynaceae	Isihlungu	Herb	Leaves and whole plant	Ornamental and herbal medicine (cancer and diabetes)	0.09
*Cenchrus ciliaris* L.; AM1569	Poaceae	Phungela	Grass	Whole plant	Fodder	0.01
*Centella asiatica* (L.) Urb.; AM1570	Apiaceae	Udingu	Climber	Leaves	Herbal medicine (skin diseases (acne) and wound)	0.02
*Centella coriacea* Nannf.; AM1453	Apiaceae	Unongotyozana	Herb	Leaves	Leafy vegetable and herbal medicine (EVM, STIs, TB and wounds)	0.10
*Cereus jamacaru DC.; AM1571	Cactaceae	Unoroshe	Shrub	Stems	Fodder	0.01
*Chenopodium album L.; AM1454	Amaranthaceae	Iphunga	Herb	Leaves	Leafy vegetable	0.11
*Cheilanthes hirta* Sw.; AM1455	Sinopteridaceae	Ifense	Pteridophyte	Leaves	Herbal medicine (wounds)	0.01
*Citrus limon (L.) Burm. f.; AM1530	Rutaceae	Lamuni	Tree	Fruits and leaves	Edible fruits, herbal medicine (skin rash) and bartered with neighbours or sold in local markets	0.19
*Citrus sinensis (L.) Osbeck; AM1529	Rutaceae	Iorenji	Tree	Fruits	Edible fruits and bartered with neighbours or sold in local markets	0.20
*Clivia miniata* Regel; AM1434	Amaryllidaceae	Umayime	Herb	Leaves	Herbal medicine (stomach problems)	0.04
*Colocasia antiquorum* Schott; AM1572	Araceae	Idumbe	Herb	Tubers	Edible tubers	0.01
*Combretum erythrophyllum* (Burch.) Sond.; AM1528	Combretaceae	Umdubu	Tree	Leaves	Fodder	0.02
*Convolvulus sagittatus* Thumb; AM1497	Convolvulaceae	Uboqo	Herb	Roots	Herbal medicine (headache)	0.01
*Cucurbita maxima Duchesne; AM1517	Cucurbitaceae	Ithanga	Climber	Fruits	Edible fruits and bartered with neighbours or sold in local markets	0.24
*Cucurbita moschata Duchesne ex Poir.; AM1498	Cucurbitaceae	Ithanga	Climber	Fruits	Edible fruits and bartered with neighbours or sold in local markets	0.30
*Cussonia paniculata* Eckl. & Zeyh.; AM1518	Araliaceae	Umsenge	Tree	Bark and leaves	Herbal medicine (immune booster and skin diseases)	0.06
*Cussonia spicata* Thunb.; AM1488	Araliaceae	Umgezisa	Tree	Leaves	Sold as herbal medicine (EVM, immune booster and stomach problems)	0.07
*Cymbopogon nardus* (L.) Rendle; AM1426	Poaceae	Umqungu	Grass	Whole plant	Thatching grass	0.01
*Cynodon dactylon (L.) Pers.; AM1509	Poaceae	Uqaqaqa	Grass	Leaves	Fodder	0.04
*Datura stramonium L.; AM1510	Solanaceae	Umhlavuthwa, umvumbangwe and uvumbangwe,	Herb	Leaves and stems	Firewood, fodder and herbal medicine (asthma, boils and wounds)	0.07
*Daucas carota L.; AM1487	Apiaceae	Kharothi	Herb	Taproots	Edible taproot and bartered with neighbours or sold in local markets	0.27
*Dicerothamnus rhinocerotis* (L.f.) Koekemoer; AM1573	Asteraceae	Umlingatho	Herb	Leaves and roots	Herbal medicine (stomach problems and fumigant)	0.01
*Dicoma capensis* Less; AM1574	Asteraceae	Ucelezi	Herb	Leaves	Herbal medicine (respiratory infections)	0.02
*Digitaria eriantha* Steud.; AM1575	Poaceae	Ulozana	Grass	Whole plant	Fodder	0.01
*Diospyros lycioides* Desf.; AM1427	Ebenaceae	Umbhongisa	Shrub	Fruits, leaves and stems	Edible fruits, firewood and fodder	0.02
*Dovyalis caffra* (Hook. f. & Harv.) Hook. f.; AM1486	Salicaceae	Incagolo	Shrub	Fruits	Edible fruits	0.04
*Dovyalis rhamnoides* (Burch. ex DC.) Burch. ex Harv. & Sond.; AM1576	Salicaceae	Umkhamgwinqi	Shrub	Fruits	Edible fruits	0.04
*Elegia tectorum* (L.f.) Moline and H.P. Linder; AM1577	Restionaceae	–	Herb	Whole plant	Construction material and thatching	0.01
*Elephantorrhiza elephantina* (Burch.) Skeels; AM1425	Fabaceae	Intolwane	Shrub	Rhizomes	Sold as herbal medicine (EVM, high blood pressure, haemorrhoids, rashes and purify blood)	0.17
*Eucalyptus camaldulensis Dehnh.; AM1485	Myrtaceae	–	Tree	Stems and leaves	Construction timber, firewood, windbreak and herbal medicine (cough and TB)	0.05
*Eucalyptus grandis W. Hill ex Maiden; AM1519	Myrtaceae	–	Tree	Stems	Construction timber, firewood and windbreak	0.03
*Euphorbia ingens* E. Mey. ex Boiss; AM1520	Euphorbiaceae	Intsema	Shrub	Latex	Herbal medicine (cancer and skin rash)	0.04
*Ficus carica L.; AM1484	Moraceae	ikwiwane	Tree	Fruits	Edible fruits	0.05
*Foeniculum vulgare Mill.; AM1578	Apiaceae	Imboziso	Herb	Leaves	Culinary herb and herbal medicine (stomach problems)	0.01
*Grewia occidentalis* L.f.; AM1526	Malvaceae	Umgaqombo	Shrub	Fruits and roots	Edible fruits and herbal medicine (EVM and wounds)	0.01
*Gunnera perpensa* L.; AM1525	Gunneraceae	Iphuzi, iphuzi lomlambo and uxobo	Herb	Rhizomes	Sold as herbal medicine (EVM, cancer, constipation, induce or augment labour, inflammation, menstrual pain and wounds)	0.18
*Harpephyllum caffrum* Bernh.; AM1483	Anacardiaceae	Umgwenya, umgwenye, umgwenye-hangul and umgwenyobomvu	Tree	Bark and fruits	Edible fruits and herbal medicine (rash and wounds)	0.12
*Harrisia balansae (K. Schum.) N.P. Taylor & Zappi; AM1579	Cactaceae	Ukatyi	Shrub	Fruits	Edible fruits	0.01
*Helichrysum gymnocomum* DC*.;* AM1458	Asteraceae	Icholachola	Herb	Whole plant	Herbal medicine (colds and cough)	0.06
*Helichrysum nudifolium* (L.) Less.; AM1457	Asteraceae	Icholocholo and insicwe	Herb	Leaves	Spiritual and herbal medicine (cough, diabetes, menstrual problems and circumcision wounds)	0.09
*Helichrysum odoratissimum* (L.) Sweet; AM1456	Asteraceae	Iphepho	Shrub	Whole plant	Spiritual and sold as herbal medicine (colds, diabetes, headache, wounds, fumigant and insect repellent)	0.12
*Helichrysum pedunculatum* Hilliard & B.L. Burtt; AM1580	Asteraceae	Iphepho	Shrub	Whole plant	Spiritual and circumcision wounds	0.03
*Hermannia depressa* N.E.Br.; AM1435	Malvaceae	Phate eangaka	Shrub	Leaves	Herbal medicine (colds and cough)	0.04
*Hyparrhenia hirta* (L.) Stapf; AM1482	Poaceae	Umngcele	Grass	Whole plant	Thatching grass	0.04
*Hypoxis argentea* Harv. ex Baker; AM1499	Hypoxidaceae	Inongwe, isinana and ixalanxa	Herb	Bulb	Herbal medicine (EVM, cancer, ringworm and TB)	0.06
*Hypoxis hemerocallidea* Fisch. Mey. & Ave-Lall.; AM1524	Hypoxidaceae	Inongwe	Herb	Bulb	Sold as herbal medicine (cancer, diabetes, high blood pressure, immune booster and pimples)	0.33
*Ilex mitis* (L.) Radlk*.;* AM1480	Aquifoliaceae	Isidumo, umduma, unduduma and unduma	Tree	Bark	Sold as herbal medicine (sore throat and stomach problems)	0.08
*Ipomoea batatas (L.) Lam.; AM1428	Convolvulaceae	Bhatata	Climber	Tubers	Edible tubers and bartered with neighbours or sold in local markets	0.21
*Lactuca sativa L.; AM1471	Asteraceae	Ilethasi	Herb	Leaves	Leafy vegetable and bartered with neighbours or sold in local markets	0.14
*Lasiosiphon capitatus* (Lam.) Burtt Davy; AM1527	Thymelaeaceae	Isidikili	Shrub	Roots	Herbal medicine (ringworm and wounds)	0.04
*Leonotis leonurus* (L.) R. Br.; AM1523	Lamiaceae	Imvovo, Umfinafincane and utywala bengcungcu	Shrub	Leaves	Herbal medicine (EVM, colds, cough, diarrhoea and snakebite)	0.09
*Lippia javanica* (Burm. f.) Spreng.; AM1479	Verbanaceae	Inzinziniba	Shrub	Leaves and roots	Herbal medicine (chicken pox, colds, cough and wounds)	0.12
*Lobelia flaccida* (C. Presl) A. DC.; AM1522	Campanulaceae	Itshilizi and ubulawu	Herb	Leaves	Herbal medicine (abdominal pain)	0.02
*Lycopersicon esculentum Mill.; AM1521	Solanaceae	Tumata	Climber	Fruits	Edible fruits and bartered with neighbours or sold in local markets	0.22
*Malus domestica Borkh.; AM1481	Rosaceae	Apile	Tree	Fruits	Edible fruits	0.10
*Malva parviflora* L.; AM1482	Malvaceae	Ujongelana and unomolwana	Shrub	Leaves	Herbal medicine (wounds)	0.01
*Mentha longifolia* (L.) Huds.; AM1483	Lamiaceae	Inxina	Herb	Leaves	Culinary herb and herbal medicine (wounds)	0.05
*Miscanthus capensis* (Nees) Andersson; AM1478	Poaceae	Idobo	Grass	Leaves and roots	Thatching grass and herbal medicine (wounds)	0.04
*Musa X *paradisiaca* L.; AM1503	Musaceae	–	Tree	Fruits	Edible fruits sold in local markets	0.13
*Nicotiana glauca Graham; AM1502	Solanaceae	Icubamfene	Shrub	Leaves	Herbal medicine (headache)	0.05
*Nidorella ivifolia* (L.) J.C.Manning & Goldblatt; AM1581	Asteraceae	Isavu	Shrub	Leaves	Herbal medicine (diabetes)	0.03
*Opuntia engelmannii Salm-Dyck ex Engelm.; AM1582	Cactaceae	Unochwane	Shrub	Stems	Fodder	0.02
*Opuntia ficus-indica (L.) Mill.; AM1501	Cactaceae	Itolofiya	Tree	Fruits, stems and whole plant	Edible fruits and bartered with neighbours or sold in local markets, fodder, live fence, animal enclosure, ornamental and herbal medicine (wounds)	0.34
*Opuntia monacantha Haw.; AM1583	Cactaceae	Tolofiya	Shrub	Fruits	Edible fruits	0.01
*Pentanisia prunelloides* (Klotzsch ex Eckl. & Zeyh.) Walp. ssp. *prunelloides;* AM1411	Rubiaceae	Cimamlilo, icimamlilo, icishamlilo, irubuxa and isicimamlilo	Herb	Roots	Herbal medicine (wounds)	0.04
*Persea americana Mill.; AM1459	Lauraceae	–	Tree	Fruits and seed paste	Edible fruits and herbal medicine (ringworm)	0.12
*Phaseolus vulgaris L.; AM1460	Fabaceae	Mbotyi	Herb	Fruits and seeds	Edible fruits and seeds	0.20
*Phoenix reclinata* Jacq.; AM1469	Arecaceae	Idama and isundu	Tree	Leaves and whole plant	Crafts and ornamental	0.04
*Phragmites australis* (Cav.) Steud.; AM1500	Poaceae	Ingcongolo	Grass	Leaves and stems	Thatching grass, building walls and animal enclosure	0.04
*Physalis angulata L.; AM1470	Solanaceae	Iyoli	Herb	Leaves	Herbal medicine (burns)	0.05
*Phytolacca dioica L.; AM1584	Phytolaccaceae	Umvumvu	Tree	Leaves and whole plant	Ornamental, fodder and shade	0.01
*Pinus halepensis Mill.; AM1585	Pinaceae	Ipayina	Tree	Whole plant	Firewood, ornamental and windbreak	0.01
*Pisum sativum L.; AM1461	Fabaceae	Erityisi	Herb	Fruits	Edible fruits sold in local markets	0.16
*Pittosporum viridiflorum* Sims.; AM1420	Pittosporaceae	Umgqwengqwe and umkhwenkwe	Shrub	Bark and roots	Herbal medicine (EVM, abdominal pain, cancer and fever)	0.12
*Plectranthus ambiguus* (Bolus) Codd; AM1429	Lamiaceae	Irhajojo	Herb	Leaves	Herbal medicine (EVM, colds and cough)	0.06
*Podocarpus latifolius* (Thunb.) R. Br. ex Mirb.; AM1511	Podocarpaceae	Umcheya	Tree	Leaves	Herbal medicine (EVM and wounds)	0.01
*Pontederia crassipes Mart.; AM1586	Pontederiaceae	Inyibiba	Herb	Whole plant	Ornamental and erosion control	0.12
*Pontederia cordata L. var. *ovalis* Solms; AM1587	Pontederiaceae	Ingcongolo	Herb	Whole plant	Ornamental and erosion control	0.01
*Portulaca oleracea* L.; AM1588	Portulacaceae	Igwanisha	Shrub	Leaves and stems	Leafy vegetable	0.01
*Portulacaria afra* Jacq.; AM1589	Didiereaceae	Igqwanitsha, igwanisha, igwanishe, umfayisele and wehlathi	Shrub	Leaves and stems	Leafy vegetable	0.01
*Prunus africana* Hook.; AM1467	Rosaceae	Inyazangoma, itywina-elikhul, umdumizulu, umkhakhase and umkhakhazi	Tree	Bark and leaves	Herbal medicine (cough, eye problems and TB)	0.04
*Prunus armeniaca L.; AM1415	Rosaceae	–	Tree	Fruits	Edible fruits	0.12
*Prunus persica (L.) Batsch; AM1512	Rosaceae	Ipesika	Tree	Fruits, leaves and stems	Edible fruits, firewood and herbal medicine (eye problems)	0.13
*Psidium guajava L.; AM1513	Myrtaceae	Gwava	Shrub	Fruits, leaves and stems	Edible fruits and bartered with neighbours or sold in local markets, and herbal medicine (diarrhoea)	0.18
*Punica granatum L.; AM1591	Lythraceae	Rhanati	Tree	Fruits, leaves and stems	Edible fruits, firewood and fodder	0.01
*Rhoicissus digitata* (L.f.) Gilg & Brandt; AM1468	Vitaceae	Uchithibhunga	Climber	Roots	Herbal medicine (headache and high blood pressure)	0.07
*Ricinus communis L.; AM1466	Euphorbiaceae	Umhlakuza and umkakuva	Tree	Leaves	Herbal medicine (stomachache)	0.05
*Rubus fruticosus L.; AM1590	Rosaceae	Qunube	Shrub	Fruits	Edible fruits and wine production	0.01
*Rumex lanceolatus* Thunb.; AM1592	Polygonaceae	Idololenkonyane and idolonyana	Shrub	Roots	Herbal medicine (EVM, back pain and rheumatism)	0.01
*Salix babylonica L.; AM1593	Salicaceae	Umngcunube	Tree	Whole plant	Ornamental	0.01
*Salvia scabra* L. f.; AM1416	Lamiaceae	Isicakathi	Herb	Leaves	Herbal medicine (EVM and tonic)	0.01
*Schotia latifolia* Jacq.; AM1418	Fabaceae	Umaphipha and umgxam	Tree	Bark and stems	Construction timber and herbal medicine (EVM and diarrhoea)	0.04
*Senecio speciosus* Willd.; AM1419	Asteraceae	Idambiso	Herb	Leaves	Herbal medicine (inflammations and wounds)	0.05
*Senegalia caffra* (Thunb.) P.J.H.Hurter & Mabb.; AM1504	Fabaceae	Umnyamanzi and umthole	Tree	Stems	Construction timber	0.04
*Sida rhombifolia* L.; AM1465	Malvaceae	Umdizawethafa	Shrub	Leaves	Herbal medicine (wounds)	0.01
*Solanum aculeastrum* Dun.; AM1417	Solanaceae	Umthuma	Shrub	Fruits	Edible fruits and herbal medicine (boils, cancer, dysentery and impotence)	0.05
*Solanum nigrum L.; AM1430	Solanaceae	Umsobo and umsobosobo	Herb	Fruits and leaves	Edible fruits and herbal medicine (ringworm and wounds)	0.07
*Solanum tuberosum L.; AM1444	Solanaceae	Amazambane	Herb	Tubers	Edible tubers and bartered with neighbours or sold in local markets	0.29
*Sonchus asper (L.) Hill; AM1445	Asteraceae	Irwabe	Herb	Leaves	Leafy vegetable	0.04
*Sonchus oleraceus L.; AM1446	Asteraceae	Ihlaba	Herb	Leaves	Leafy vegetable	0.05
*Spinacia oleracea L.; AM1412	Amaranthaceae	Imifuno	Herb	Leaves	Leafy vegetable and bartered with neighbours or sold in local markets	0.54
*Sporobolus africanus* (Poir.) Robyns & Tournay; AM1431	Poaceae	Umtshiki	Grass	Whole plant	Thatching grass	0.04
*Sporobolus fimbriatus* (Trin.) Nees; AM1436	Poaceae	Umgigwi	Grass	Whole plant	Thatching grass	0.03
*Syzygium cordatum* Hochst. ex C. Krauss.; AM1443	Myrtaceae	Umswi	Tree	Bark	Herbal medicine (inflammation and pimples)	0.06
*Syzygium paniculatum Gaertn.; AM1594	Myrtaceae	Irharinati	Tree	Fruits	Edible fruits	0.01
*Tagetes minuta L.; AM1595	Asteraceae	Nnkayo	Herb	Whole plant	Herbal medicine (anthelmintic, insect repellent and stomach problems)	0.02
*Taraxacum officinale Weber; AM1463	Asteraceae	Ikhokhoyi	Herb	Leaves	Leafy vegetable	0.05
*Tecoma capensis* (Thunb.) Lindl.; AM1596	Bignoniaceae	Umsilingi	Tree	Leaves	Herbal medicine (inflammation and pain)	0.01
*Toxicodendron succedaneum (L.) Kuntze; AM1597	Anacardiaceae	Gamtriya	Tree	Leaves	Herbal medicine (wounds)	0.01
*Trichilia emetica* Vahl; AM1437	Meliaceae	Isibara and umkhulu	Tree	Leaves	Herbal medicine (wounds)	0.04
*Tulbaghia acutiloba* Harv*.*; AM1462	Amaryllidaceae	Isivumbampunzi	Herb	Leaves	Herbal medicine (colic)	0.04
*Tulbaghia alliacea* L. f.; AM1442	Amaryllidaceae	Umwelela	Herb	Bulb	Herbal medicine (boils and wounds)	0.06
*Tulbaghia violacea* Harv.; AM1464	Amaryllidaceae	Utswelane	Herb	Bulb	Herbal medicine (cancer and TB)	0.07
*Typha capensis* (Rohrb.) N. E. Br.; AM1438	Typhaceae	Ingcongolo and umkhanzi	Herb	Leaves and rhizomes	Construction material, crafts, green mature, water purification and herbal medicine (dysentery and STI)	0.07
*Vachellia karroo* (Hayne) Banfi & Glasso; AM1423	Fabaceae	Umnga, umnga-mpunzi and umunga	Tree	Bark, leaves and stems	Construction timber, firewood, fodder and sold as herbal medicine (EVM, boils, diarrhoea, haemorrhage, ringworm, thrush and TB)	0.35
*Vitis vinifer L.; AM1440	Vitaceae	Umdiliya	Climber	Fruits	Edible fruits sold in local markets	0.06
*Withania somnifera* (L.) Dunal; AM1414	Solanaceae	Ubuvuma	Shrub	Leaves and roots	Herbal medicine (inflammations, TB and wounds)	0.04
*Xanthium spinosum L.; AM1598	Asteraceae	Itshungu	Herb	Roots	Herbal medicine (wounds)	0.03
*Xysmalobium undulatum* (L.) W.T. Aiton; AM1442	Apocynaceae	Ishongwane, itshongwe, iyeza elimhlophe and nwachaba	Herb	Roots	Herbal medicine (STIs)	0.05
*Zantedeschia aethiopica* (L.) Spreng.; AM1537	Araceae	Inyiba	Herb	Leaves	Sold as herbal medicine (wounds)	0.04
*Zea mays L.; AM1441	Poaceae	umbone	Grass	Fruits	Edible seeds and fruits and bartered with neighbours or sold in local markets	0.57
*Ziziphus mucronata* Willd.; AM1413	Rhamnaceae	Umphafa	Tree	Leaves	Herbal medicine (EVM, chest pains, cough, dysentery and TB)	0.11

Species marked with an asterisk (*) are exotic to South Africa

**Table 2:** Plant families of utilized plant species with the largest number of species (with at least 3 species)

Family	Number of species	%
Asteraceae	16	9.8
Poaceae	14	8.6
Fabaceae	10	6.1
Solanaceae	9	5.5
Amaryllidaceae	8	4.9
Asphodelaceae	7	4.3
Apiaceae	6	3.4
Amaranthaceae	5	3.1
Asparagaceae	5	3.1
Cactaceae	5	3.1
Myrtaceae	5	3.1
Rosaceae	5	3.1
Apocynaceae	4	2.5
Malvaceae	4	2.5
Lamiaceae	4	2.5
Salicaceae	3	1.8

### Ecosystem services identification

**Fig. 2:**
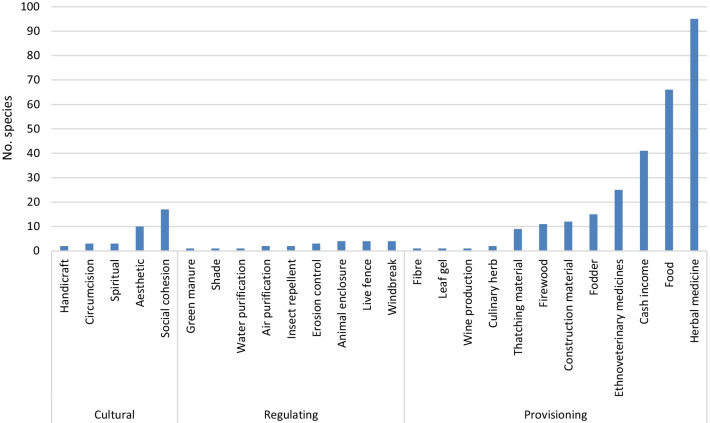
Identification of ecosystem services

A total of five cultural services (aesthetic, circumcision ritual, handicrafts, spiritual, social cohesion and integration), nine regulating services (air purification, animal enclosure, erosion control, green manure, insect control, live fencing, shading, windbreak and water purification) and 12 provisioning services (cash income, construction materials, culinary herbs, ethnoveterinary medicines, fibre, firewood, fodder, food, herbal medicines, leaf gel, thatching materials and wine production) were identified through interviews and observations made during field work (Fig. 2). Traditional male circumcision is an important cultural ritual practiced by the Xhosa people in the Eastern Cape Province. Male circumcision is carried out for cultural reasons, as an initiation ritual and a rite of passage or transition from boyhood to manhood [84–86]. The foreskin is cut off without anaesthesia and the wound is not stitched but bound in traditional medicines to help in the healing process [84–86]. The majority of species recorded in this study provided provisioning services such as herbal medicines (95 species, 58.3%), food (67 species, 41.1%), source of income (41 species, 25.2%), ethnoveterinary medicines (25 species, 15.3%), fodder (15 species, 9.2%), construction materials (12 species, 7.4%), firewood (11 species, 6.7%) and thatching materials (9 species, 5.5%) (Fig. 2). The cultural and regulating services were characterized by lower number of plant species than provisioning services. This result is consistent with previous studies that identified provision of food, medicinal products, construction materials, firewood, fibre and fodder as the most important ESs provided by wild and cultivated plant species [87–90]. Research by Landreth and Saito [91] showed that ESs derived from plant resources are diverse and subject to environmental, economic and cultural livelihood needs. Other researchers argued that food provisioning is particularly important in rural areas of subsistence economies as this ES is important for the well-being of households [92–94]. Similarly, a study by Mensah et al. [3] carried out in the Greater Letaba Municipality in the Mopani District of the Limpopo Province in South Africa revealed the dominance of the provisioning ESs such as edible plants, firewood and timber.

**Fig. 3:**
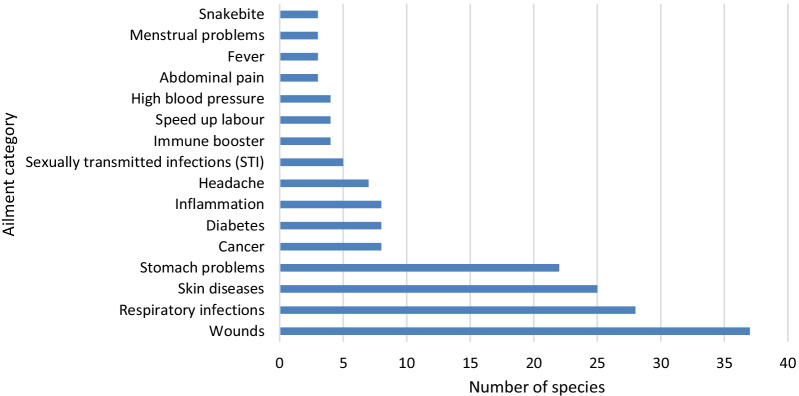
Major ailments and disease categories and number of species reported. Most species were reported in more than one ailment category

A total of 30 medical conditions were treated using remedies prepared from medicinal plants (Table 1). Wounds, respiratory infections, skin diseases, stomach problems, cancer, diabetes, inflammation, headache and sexually transmitted infections (STI) were treated with the highest number of medicinal plant species (Fig. 3). Research by Ghuman et al. [95] showed that medicinal plants are widely used in South Africa for treating wounds, eczema, ringworm, sores, boils, pimples and infected wounds. Similarly, research by Louw et al. [96] showed that monocotyledonous geophytes and bulbous plants indigenous to South Africa are characterized by valuable pharmacological properties such as the analgesic, anticancer, antimutagenic, immune stimulating, anti-infective, antimalarial, cardiovascular and respiratory system effects. Results of this study showed that the value of medicinal plants in terms of number of species traded in the Eastern Cape Province is significant as 45.9% of the traded species were medicinal species in comparison with 54.1% food plants that were bartered with neighbours or sold in local markets (Table 1). Interviews with participants showed that the value of medicinal plants is not only for primary healthcare, but financial, cultural identity and livelihood security. Moreover, previous research by Van Wyk et al. [97] showed that medicinal plants are an important aspect of the daily lives of many people and an important part of the South African cultural heritage.

**Fig. 4:**
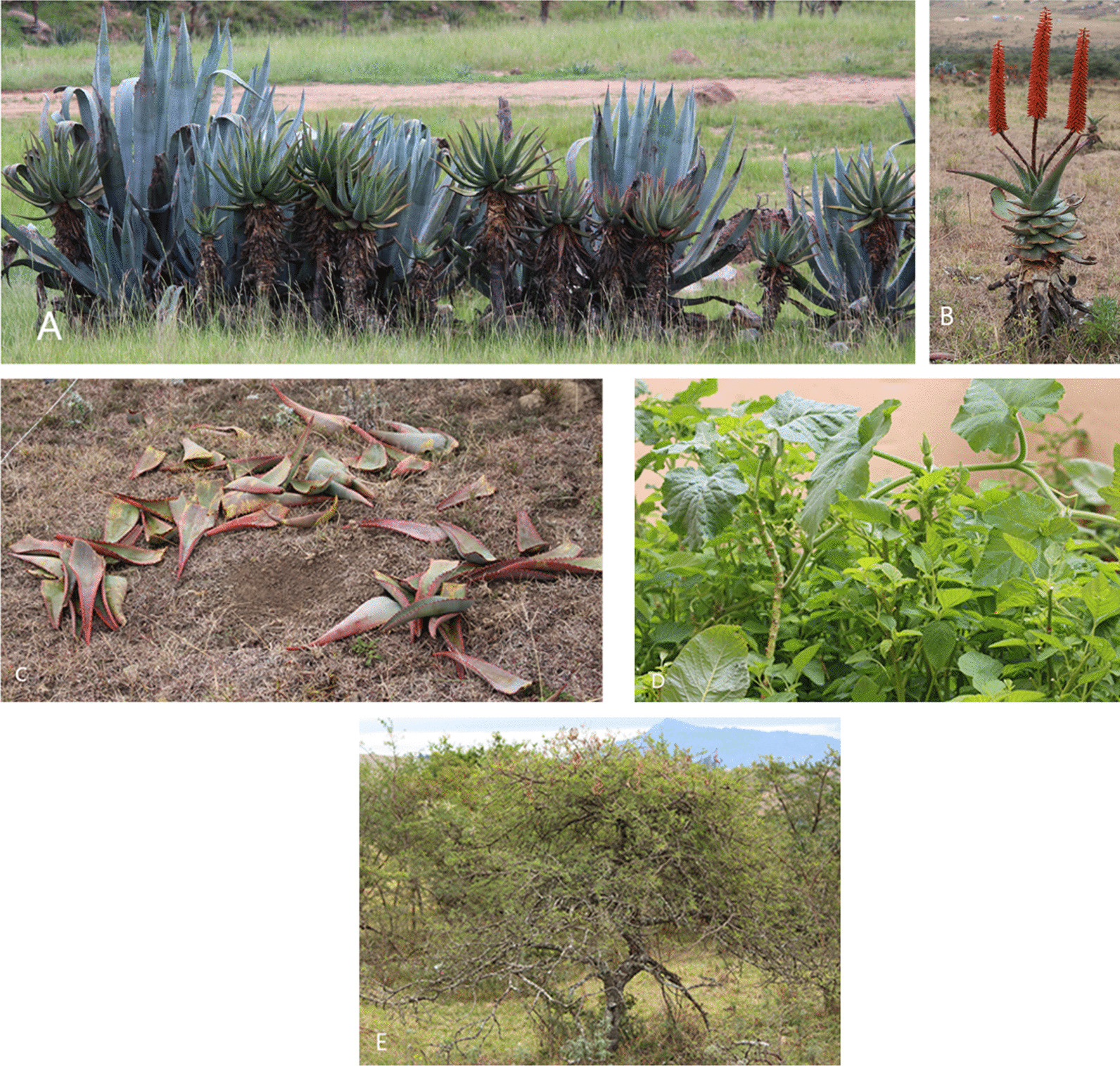
Some plant species recorded in the Eastern Cape. **A** Live fence of *Agave americana* and *Aloe ferox*, **B** Harvested *Aloe ferox*, **C** Harvested leaves of *Aloe ferox*, **D** Intercropping of *Amaranthus hybridus*, *Brassica* spp. and *Cucurbita moschata* and **E** *Vachellia karroo* (photos: Alfred Maroyi)

The number of species used for regulating services ranged from one to four species. Species such as *Aloe ferox* Mill., *Caesalpinia decapetala* (Roth) Alson, *Opuntia ficus-indica* (L.) Mill. and *Phragmites australis* (Cav.) Steud. were used to reinforce animal and/or livestock enclosures. *Agave americana* L., *Aloe ferox* (Fig. 4), *Caesalpinia decapetala* and *Opuntia ficus-indica* were used as live fence, while *Acacia mearnsii* De Wild., *Eucalyptus camaldulensis* Dehnh., *Eucalyptus grandis* W. Hill ex Maiden and *Pinus halepensis* Mill. were used as windbreak (Table 1). Plant species used for cultural services included *Helichrysum* species, that is, *H. nudifolium* (L.) Less., *H. odoratissimum* (L.) Sweet and *H. pedunculatum* Hilliard & B.L. Burtt. These three *Helichrysum* species were used as incense to invoke the goodwill of the ancestors and/or used during ceremonial events. *Boophone disticha* (L. f.) Herb., *Helichrysum nudifolium* and *Helichrysum pedunculatum* were used against circumcision wounds (Table 1). Previous research by Calvet-Mir et al. [8] revealed that cultural services are less developed in the literature on ESs although this category plays a central role in explaining the societal value of plant species to several communities around the world. Plant species characterized by edible fruits, seeds, taproot, tubers and those used as leafy vegetables were bartered with neighbours or sold in local markets, reinforcing social cohesion and integration. It was observed that plants sold in large quantities in local markets were species in high demand such as medicinal plants, fruits and leafy vegetables. *Agapanthus africanus* Hoffmanns, *Agave americana*, *Catharanthus roseus* (L.) G. Don, *Opuntia ficus-indica*, *Phoenix reclinata* Jacq., *Phytolacca dioica* L., *Pinus halepensis*, *Pontederia crassipes* Mart., *Pontederia cordata* L. var. *ovalis* Solms and *Salix babylonica* L. were grown or maintained around homesteads as ornamental or for their aesthetic value (Table 1). A similar trend was reported by Ndayizeye et al. [98] where the Twa hunters, gatherers and farmers of Burundi sold forest products such as medicinal plants, ethnoveterinary medicines, fodder, ornamental plants and wild vegetables. Therefore, wild plant species deserve special attention due to their possible role as off-farm sources of income, particularly for communities in remote and marginalized areas with limited sources of livelihoods.

**Fig. 5:**
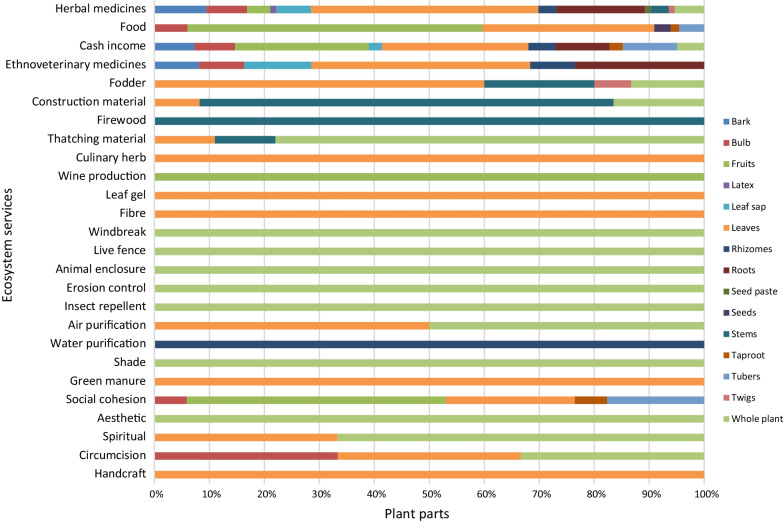
Relative contribution of plant parts towards ecosystem services. Different colours indicate specific plant parts

Figure 5 shows different plant parts utilized to provide various ESs. Whole or entire plants were associated with aesthetic plants, air purification, animal enclosures, erosion control, insect repellent, live fence, shading and windbreak (Fig. 5). Tree stems were used as firewood and water purification, while leaves were used as sources of culinary herbs, fibre, green manure, handcraft and leaf gel. Several plant parts were used as sources of herbal and ethnoveterinary medicines, food plants and cash income. However, harvesting of bark, bulbs, rhizomes, roots, stems and whole or entire plants, particularly herbaceous plants for medicinal purposes is not sustainable as such strategies threaten the survival of the same species used as food or to treat or manage human ailments or animal diseases.

Important species recorded in this study with RFC values > 0.3 included *Alepidea amatymbica* Eckl. & Zeyh., *Allium cepa* L., *Aloe ferox*, *Artemisia afra* Jacq*.* ex Willd., *Brassica oleracea* L., *Capsicum annuum* L., *Cucurbita moschata* Duchesne ex Poir., *Hypoxis hemerocallidea* Fisch. Mey. & Ave-Lall., *Opuntia ficus-indica*, *Spinacia oleracea* L., *Vachellia karroo* (Hayne) Banfi & Glasso and *Zea mays* L. *Allium cepa* (onion), *Brassica oleracea* (cabbage), *Capsicum annuum* (pepper), *Cucurbita moschata* (butternut), *Spinacia oleracea* (spinach) and *Zea mays* (maize) were widely grown as food crops assisting in the provision of necessary nutrients and food security. *Alepidea amatymbica* and *Hypoxis hemerocallidea* are categorized as threatened in South Africa [99] due to over-collection as traditional medicines [46]. Other plant species that were recorded in this study that are categorized as threatened in South Africa include *Boophone disticha* (L. f.) Herb., *Bowiea volubilis* Harv. ex Hook. f. ssp. *volubilis, Clivia miniata* Regel, *Gunnera perpensa* L., *Ilex mitis* (L.) Radlk. and *Prunus africana* [88]. It is well recognized that medicinal plants primarily valued for their medicinal properties are intensively harvested and some of them tend to be the most threatened by over-exploitation.

## Conclusion

Results of this study indicate that local communities in the Eastern Cape Province in South Africa derive ESs such as traditional medicines, food, raw materials, cultural and regulating benefits from plant species collected from the wild as well as cultivated species. This study showed that provisioning services were perceived as the most important ES in comparison with regulating and cultural services. These results highlight the importance of plant resources to the well-being of local communities in the context of provision of essential direct and indirect ESs such as food, medicinal products, construction materials, fodder, regulating, supporting and cultural services. The ESs are the basis for subsistence livelihoods in rural areas, particularly in developing countries such as South Africa. Therefore, understanding the ESs that can be derived from wild and cultivated plant species is important, as well as the implications of utilization of such natural resources in the context of rural livelihoods and well-being. These ESs place plant resources in a web formed by linkages with different ESs services derived from agricultural landscapes. Therefore, these research findings contribute to the wider body of knowledge on ESs derived from plant species, expanding the understanding of the uses and values of plant resources, the livelihood benefits derived by local communities from plant species, and how these benefits influence local support for natural resource management initiatives in the province and other areas of the country.

## Data Availability

Raw data is contained in questionnaire forms and cannot be shared in this form.
